# Limitations and modifications in the clinical application of calcium sulfate

**DOI:** 10.3389/fsurg.2024.1278421

**Published:** 2024-02-29

**Authors:** Deng-xing Lun, Si-ying Li, Nian-nian Li, Le-ming Mou, Hui-quan Li, Wan-ping Zhu, Hong-fei Li, Yong-cheng Hu

**Affiliations:** ^1^ Department of Spinal Degeneration and Oncology, Weifang People’s Hospital, Weifang City, Shandong, China; ^2^ Department of Bone Oncology, Tianjin Hospital, Tianjin, China

**Keywords:** biomineral, calcium sulfate, bone substitute materials, material processing, synthetic substitutes

## Abstract

Calcium sulfate and calcium sulfate-based biomaterials have been widely used in non-load-bearing bone defects for hundreds of years due to their superior biocompatibility, biodegradability, and non-toxicity. However, lower compressive strength and rapid degradation rate are the main limitations in clinical applications. Excessive absorption causes a sharp increase in sulfate ion and calcium ion concentrations around the bone defect site, resulting in delayed wound healing and hypercalcemia. In addition, the space between calcium sulfate and the host bone, resulting from excessively rapid absorption, has adverse effects on bone healing or fusion techniques. This issue has been recognized and addressed. The lack of sufficient mechanical strength makes it challenging to use calcium sulfate and calcium sulfate-based biomaterials in load-bearing areas. To overcome these defects, the introduction of various inorganic additives, such as calcium carbonate, calcium phosphate, and calcium silicate, into calcium sulfate is an effective measure. Inorganic materials with different physical and chemical properties can greatly improve the properties of calcium sulfate composites. For example, the hydrolysis products of calcium carbonate are alkaline substances that can buffer the acidic environment caused by the degradation of calcium sulfate; calcium phosphate has poor degradation, which can effectively avoid the excessive absorption of calcium sulfate; and calcium silicate can promote the compressive strength and stimulate new bone formation. The purpose of this review is to review the poor properties of calcium sulfate and its complications in clinical application and to explore the effect of various inorganic additives on the physicochemical properties and biological properties of calcium sulfate.

## Introduction

As the population ages, the incidence of bone defects as a result of infection, bone tumor, surgery, or major trauma is increasing significantly (1, 2), and the consequent demand for bone grafting materials is also continuously increasing. Currently, bone substitute materials have been an area of intense research interest, and a variety of materials have been used to fill bone defects and promote bone regeneration, including autografts, allografts, calcium phosphate, bioglass, calcium sulfate, and their compounds (3–12). However, each bone substitute has its own unique advantages and disadvantages (7–19).

Autologous grafts are considered the gold standard for defect reconstruction (5, 6) as they confer complete histocompatibility while possessing osteoinductive, osteoconductive, and osteogenic healing potential. However, an autologous graft has 0.76%–39% harvesting procedure-associated complications (7–9), which include increased blood loss, operative time (10), and donor site pain, as well as the potential for donor site infection, hematoma, and nerve injury (8, 11). Another limitation is an inherently limited availability (12). Allografts, including cortical, cancellous, and demineralized bone matrix (DBM), are a bone substitute alternative to autogenous bone. An allograft exhibits osteoconductive and sometimes osteoinductive potential (such as DBM). However, the potential for disease transmission, immunogenic response, and quality variability makes an allograft an imperfect substitute. In particular, for DBM, the osteoinductivity of the allograft is greatly dependent on its preparation technique (13). Calcium phosphate, mainly tricalcium phosphate and hydroxyapatite (HA), has shown favorable results. In particular, HA, which is structurally similar to the mineral phase of bone, has been the most widely employed bioceramic for hard tissue repair. However, its poor resorbability hinders the formation of new bone and obstructs bone healing (14, 15), and calcium phosphate cement hardly induces the formation of a bone-like HA layer *in vitro* and *in vivo* (16) Several previous studies have reported that tricalcium phosphate resorbs over a period lasting between 6 and 18 months (10, 17), whereas hydroxyapatite can resorb over a period ranging from 6 months to 10 years (11). Bioactive glasses possess excellent osteoconductivity and the ability to stimulate bone formation, while their high brittleness and poor resorbability limit their application in load-bearing sites (18, 19).

Given the aforementioned complications, calcium sulfate (CS) has attracted much attention due to its unique physicochemical and biological properties, which not only provide many advantages over other types of bone graft materials, such as biocompatibility, osteoconductivity, and biodegradability (11), but also are very effective at delivering high levels of local antibiotics or drugs due to complete degradation *in vivo* (20). In addition, calcium sulfate with injectability and moldability can fill bone defects with irregular shapes and various sizes because it can be inserted in line with the shape of a defective region (21). The main drawbacks of CS are its low mechanical strength and fast absorption, which means it is unable to provide any significant long-term mechanical support for vertebral compression fracture or use in load-bearing as a structural bone graft (22). The early use of pure calcium sulfate, known as the “plaster of Paris”, showed the disadvantage of delayed wound healing and osteolytic processes during rapid resorption by pH lowering (23). The side effects caused by poor properties should be sufficiently considered in the preparation of materials or clinical applications (12).

**Figure 1:**
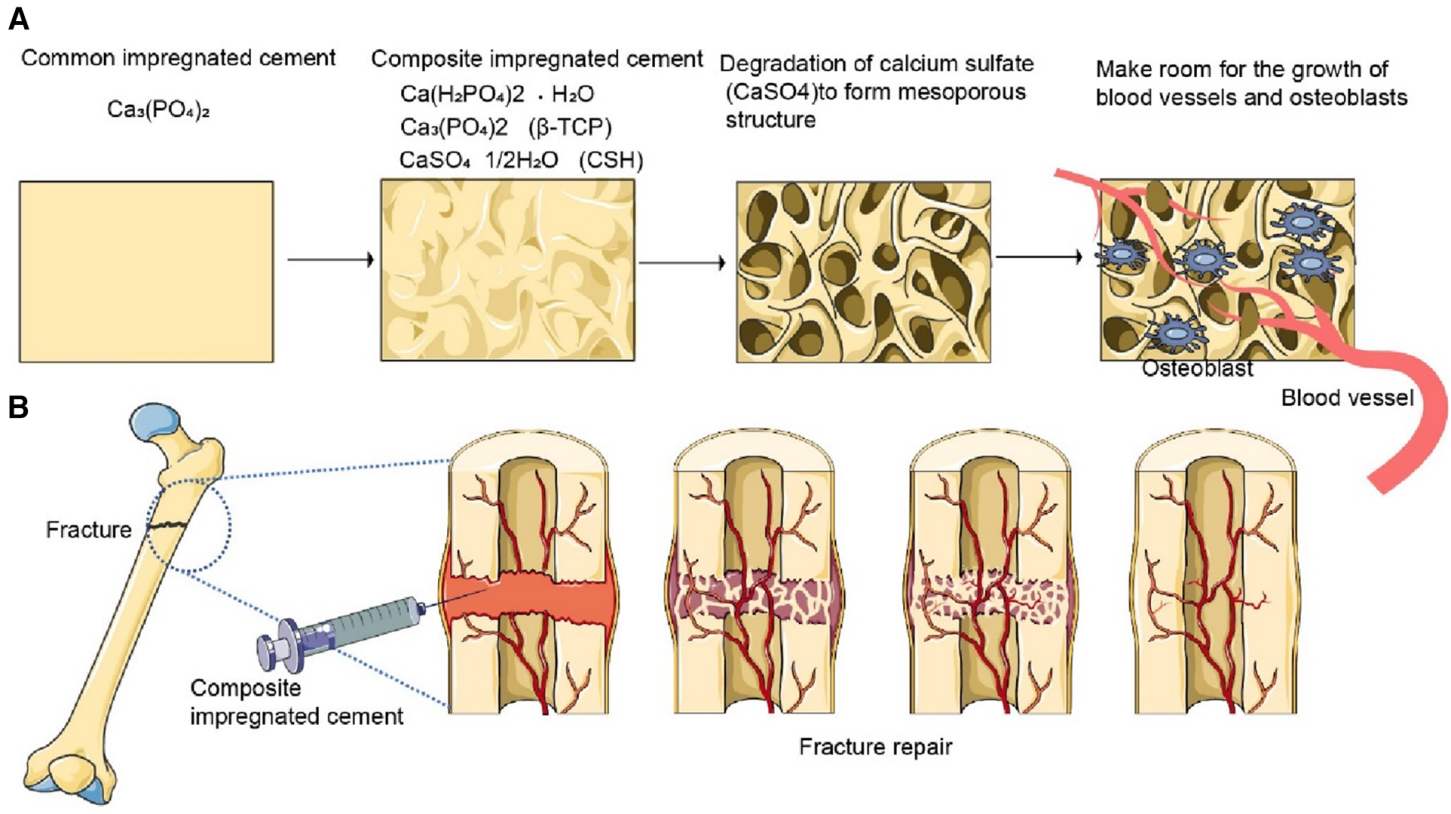
Repair effect of bone cement in bone injury. (**A**) The original bone cement is mainly composed of calcium phosphate, which mainly plays a supporting role and has poor absorption, requiring surgical removal. The commonly used composite bone cement now consists of calcium dihydrogen phosphate, calcium sulfate, and calcium sulfate hemihydrate. Calcium sulfate hemihydrate is easily degraded to form mesopores, leaving room for bone cell growth and vascular growth. (**B**) After a bone injury occurs, the bone cement composite is injected into the injury site. The bone cement supports the growth of blood vessels and bone cells. After the wound is repaired, the bone cement is completely absorbed.

To mitigate implant-related complications in clinical applications, it is of great significance for clinicians and researchers to understand the relationship between the inherent limitations of calcium sulfate and its complications in clinical application (24). Previous studies reported that the complications caused by calcium sulfate may be related to absorption that is too fast. For example, many sulfate ions released into surrounding tissue during degradation result in wound drainage, and large amounts of calcium ions result in hypercalcemia. Therefore, considerable efforts have been made to improve the physical and chemical properties of CS by the addition of a substance, e.g., calcium phosphate or calcium silicate (23) (Figure 1).

Currently, few studies have reported the mechanism of the problems mentioned above caused by the poor properties of calcium sulfate and the modified methods to prevent these complications. Therefore, the aim of this study was to summarize the insufficient properties of calcium sulfate and consequent complications and to elaborate the interaction mechanism. Another purpose was to investigate the effect of inorganic salt additives on the compressive strength and degradation rate to design and fabricate more perfect calcium sulfate-based materials for researchers.

## Rapid degradation rate

An ideal bone graft substitute should have the same speed of degradation as the formation of new bone tissue (25). Absorption that is too slow will hinder cell penetration into the implants and impede bone remodeling (26, 27), while absorption that is too fast will create a gap between native bone tissue and implanted material (28), which is very harmful to the reconstruction of the bone defect (29). Therefore, the degradation rate of bone graft substitutes is a critical design parameter for bone tissue regeneration, exerting a crucial impact on the early-stage and long-term performance of bone tissue repair (30).

Resorption of calcium sulfate usually occurs faster than bone formation (31)^.^ Previous studies reported that pure calcium sulfate resorbs over a period of 4–6 weeks in a contained osseous defect (31–34). Petruskevicius et al. (35) do not recommend calcium sulfate for implantation in humans, as 6 weeks is too short a time for complete degradation. The main reason for rapid resorption is the absence of a strong chemical bond between the calcium sulfate particles, which makes it easy for them to be dissolved by tissue fluid. The property of rapid resorption has an adverse effect on results in clinical application, which can decrease new bone growth or fusion rate (36), reduce mechanical stability (37), and increase implant-related complications by releasing large amounts of hydrogen and calcium ions into surrounding tissue early after implantation (36, 37). Glazer PA et al. (36) investigated the degradation rate and fusion rate of CS as a bone graft substitute in promoting spine fusion in a rabbit model and found that no fusions were observed in the group containing only calcium sulfate because it was completely absorbed within 4 weeks after surgery.

The degradation mechanism of calcium sulfate may be related to the process of dissolution *in vivo* and subsequent cellular phagocytosis (38–41). Therefore, to fabricate calcium sulfate with a controllable absorption rate, a possible method is to mix inorganic materials with a low dissolution rate, which can adjust the overall degradability by controlling the proportion of additives (42). At present, the commonly used inorganic additives include calcium carbonate, calcium silicate, and calcium phosphate. The mechanism by which calcium carbonate reduces the degradation rate of calcium sulfate is not clear. The early hypothesis was that calcium carbonate adheres to the surface of calcium sulfate to form agglomerates, which have a low dissolution rate in body fluids (43, 44). Dewi AH et al. (43) investigated the effect of the addition of calcium carbonate on the degradation performance and new bone formation of CS *in vitro* and *in vivo* and found that the incorporation of calcium carbonate into CS can decrease the degradation rate of the cements and induce faster bone formation *in vitro*. In vivo, a rat femoral defect model was used to further observe the degradation and osteogenesis of the calcium sulfate and calcium carbonate complex, and the results demonstrated that the addition of CaCO_3_ not only reduced the degradation rate of CS but also increased the initial bone formation. The author considered that combined CS and CaCO_3_ is a promising bone graft substitute. D. Pförringer et al. (44) further verified the effect of the addition of calcium carbonate on the degradation performance of CS in a rabbit model and found that calcium sulfate hemihydrate (CSH) containing calcium carbonate revealed slow and similar degradation from the 4th to the 12th week, with well-distinguishable implants at 12 weeks, while CSH alone was subject to quick degradation, which was only faintly detectable after the 4th week and had already disappeared radiologically after 6 weeks. The author concluded that the addition of calcium carbonate makes CS more resistant to resorption, leaving new bone time to form during a prolonged degradation process. At present, there are commercially available composite products of calcium sulfate and calcium carbonate, Herafill from Germany, which are used mainly as antibiotic carriers in the treatment of periprosthetic infection (44, 45). In addition, calcium sulfate incorporated with calcium carbonate has also been shown to stimulate bone regeneration in an animal model (43). However, further analyses are needed to determine whether it is effective in patients with uninfected bone defects.

The mechanism of calcium silicate decreasing the absorption rates of calcium sulfate-based compounds is that calcium silicate hydrate, a product of the reaction between calcium silicate and water, has a significantly lower dissolution rate than calcium sulfate dihydrate and in turn decreases the degradation rate of the composite materials. Moreover, the adherence of calcium silicate hydrate particles on the surfaces of calcium sulfate dihydrate crystals could reduce the contact of calcium sulfate dihydrate crystals with the soaking solution, thereby resulting in a decrease in dissolution. Huan et al. (44) investigated the effect of tricalcium silicate on the degradability of composite cements and found that the higher the percentage of tricalcium silicate was, the lower the degradation rate. It took 7 days to be completely absorbed for pure CSH, 10 days for 20% tricalcium silicate, and 21 days for 30% and 40% tricalcium silicate. In addition, the compressive strength increased with the addition of tricalcium silicate. Multiple studies have shown that tricalcium silicate can combine with various substances to improve the compressive performance of composite bone repair materials. For example, Chen et al. (45) found that the compressive strength of tricalcium silicate/sodium alginate is as high as 54 Mpa, while Ji et al. (48) successfully fabricated novel bioactive organic–inorganic composite bone elements from tricalcium silicate, sodium alginate, and calcium sulfate hemihydrate. Depending on the synergistic combination of hydration and gelation, composite cements (45/45/10 wt%) obtained extremely higher compressive strength by up to 92.41 MPa as compared with each single component. A result from a previous study by Hao et al. (49) showed that the percentage of residual material for cement with 40% tricalcium silicate was 69.33% at the 8th week and 47.16% at the 12th week after implantation, whereas the pure CSH was resorbed entirely at the 8th week. The author concluded that the reason for the lower degradation rate of composite cement than pure CS was due to the presence of tricalcium silicate, which has moderate degradability. In addition, Chen et al. (50) found that the addition of dicalcium silicate had the ability to decrease the degradation rate of CSH. The pure CSH cement degraded completely by 90 days, while the degradation rates of CSH with 20%, 40%, 60%, 80%, and 100% CSH were still as high as 60%, 50%, 41%, 29%, and 12% after 180 days of the soaking experiment, respectively, which indicated a significant difference. In short, these results show that calcium silicate can significantly reduce the degradation rate of calcium sulfate, and the degradation rate decreases with increasing calcium silicate concentration. At present, there is still no commercially available product of the calcium silicate/calcium sulfate complex. However, the advantages of calcium silicate make it one of the more popular composite materials.

Calcium phosphate has good osteogenic and biocompatibility and can be prepared into different forms (51). Compared to CS, α-Tricalcium phase(α-TCP) has injectability, plasticity, self-setting, and a slow degradation rate (52). Calcium phosphate is the first inorganic salt material used in the preparation of calcium sulfate-based products because of its slower absorption performance. Whether calcium phosphate can reduce the degradation rate of calcium sulfate remains controversial. Researchers have attempted to combine calcium phosphate and CS to prepare bone cement with an appropriate degradation rate and maintain a balance with the formation rate of new bone (4). Cheng et al. (53) successfully developed a novel type of tricalcium phosphate/calcium sulfate particles, which are injectable, biocompatible, osteogenic, and *in situ* self-fixing two-phase particles capable of forming macroporous scaffolds. Xu et al. (54) mixed silk fiber nanofibers (SFF) or deionized water with α-CSH and calcium sulfate dihydrate (CSD), and α-TCP solid phase was mixed in different proportions to prepare composite materials. When the α-TCP content is less than 25%, SFF and the addition of α-TCP can reduce the degradation rate of bone cement from 98.6 ± 2.7% to 55.5 ± 14.1%. Yang et al. (55) reported the *in vivo* performance of injectable biphasic synthetic bone graft material composed of calcium sulfate and β-tricalcium phosphate. Their results revealed that new bone formation was detected with an appropriate material resorption after 8 weeks into the sheep vertebral bone defect model. Nadkarni et al. (46) reported that composites of calcium sulfate with calcium phosphate can be formulated to resorb at controlled rates. A composite containing 35% of the weight of calcium phosphate showed that 33.3% of the implant volume was resorbed after 3 weeks in femoral metaphyseal defects in rabbits, while after 6 weeks, the resorption was 51.28% (56). An *in vitro* dissolution study indicated that 90% of the implant material was resorbed after 7 days for the pure CaSO_4_ controls and after 24 days for the CaSO_4_/CaPO_4_ composite graft samples (57) (Figure 1).

As documented, the ideal absorption time of the material should be 12–16 weeks, requiring a moderate and homogeneous degradation performance in equilibrium with the bone healing process (58). It is believed that the degradation time of calcium sulfate can reach this ultimate goal by adjusting the type and dosage of additives.

## Compressive strength

Compressive strength is a crucial factor in assessing the excellent performance of calcium sulfate bone materials (59, 60). Bone materials with high compressive strength exhibit advantages such as high strength and robust durability, making them more suitable for areas with heavy loads (61). However, their compressive strength is not directly proportional to their performance. Excessive compressive strength can result in heightened foreign body sensation and may even impact the natural healing of bones (62). Conversely, bone materials with low compressive strength share characteristics closer to natural bone, but they also manifest the same drawbacks. Due to limited compressive strength, they are prone to brittle fractures and diminished mechanical stability (63). Low compressive strength is one of the main defects of calcium sulfate, which limits its application in load-bearing areas. A previous study showed that the compressive strength of medical-grade injectable CS is only 2.4 MPa after 7 min of mixing and approximately 10 MPa after 1 h (22). A higher initial mechanical strength is of great significance to provide temporary structural support during remodeling (64). In addition, the mechanical properties of materials will also change greatly with the time of implantation or immersion (65), which will affect defect stability during the whole process of new bone growth. The mechanical strength of the material should be maintained for at least 4 weeks optimally *in vivo* (66). However, Chen et al. (67) reported the weight loss of calcium sulfate-based compounds at different time points after SBF soaking and found that the compressive strength of the cement decreased to 13.6 MPa after 14 days from 18.0 MPa after immersion for 1 day and then sharply dropped to 2.7 MPa after 42 days. We can infer that the mechanical properties of calcium sulfate are characterized by the inherent low compressive strength and sharp decrease of compressive strength with rapid absorption.

The mechanism of compressive strength in the conversion of hemihydrate calcium sulfate to dihydrate calcium sulfate has been elaborated. Morphological studies by Singh et al. (68) have shown that the strength developments in the hydrating hemihydrate paste are related to the interlocking structure of CSH. Finot et al. (69) found that the mechanical properties of calcium sulfate are related to interlocking structures and adhesion forces between particles during setting by atomic force microscopy. Amathieu (70) also proposed that the mechanical strength is due to an interlocking structure and intercrystalline interactions. Thus, the combined effect of the interlocking structure and the force of adhesion between gypsum crystal faces controls the mechanical properties. However, it is challenging to manufacture a high compressive strength similar to that of PMMA by relying solely on the original interlocking structure and adhesive force.

Considerable efforts have been made to improve compressive strength. Nilsson et al. (64) found that the compressive strength decreased with increasing tricalcium phosphate (TCP), reached a minimum at 20 wt% (5 MPa), and then increased linearly with the increasing weight proportion of TCP, starting from cement containing 20 wt% to cement consisting only of TCP (34 MPa). The authors deemed that the structure of interlocking needles produced the strength of calcium sulfate dihydrate, which was lost when less than 20% TCP was added. Sony et al. (71) investigated the effect of the addition of hydrogen orthophosphate on the mechanical properties of calcium sulfate paste and found that the compressive strength increased with higher concentrations of hydrogen orthophosphate, which may possibly have been driven by the decrease in particle size due to the occurrence of secondary nucleation during precipitation and the consequent tight packing. In addition, the interconnecting aggregate structures also have a significant role in increasing the compressive strength. Chang et al. (72) found that the compressive strength increased as calcium phosphate cement (CPC) was added and was strongly correlated with the weight ratio of CPC. The lowest compressive strength was that of the pure CSH cement (15.3 MPa), and the highest compressive strength was that of CPC (40.4 MPa).

In addition, calcium silicate has been shown to improve the compressive strength of calcium sulfate. Huan et al. (46) investigated the effect of tricalcium silicate on the mechanical properties of composite cements and found no significant difference in the compressive strength between CSH alone and CSH containing tricalcium silicate after the final setting time. However, a significant difference was seen after a prolonged time, and the compressive strength of CSH with 40% tricalcium silicate was 11.9 ± 0.6 MPa, which was significantly higher than the compressive strength of pure CSH (5.8 ± 0.5). Chen et al. (50) found that the incorporation of dicalcium silicate decreased the mechanical properties of CSH in a short period after the final setting time because the hydration reactions of calcium sulfate and silicic acid were inhibited by each other by mutually hindering contact with water. Thereafter, the compressive strength increased with increasing dicalcium silicate content. In addition, the authors investigated the relationship between the strength and soaking time in SBF and found that all specimens gradually lost their compressive strength with an increased soaking time. Bioglass, whose main component is silicate, has been used to improve the strength of calcium sulfate. Mehran Dadkhah et al. (73) compared the compressive strengths of calcium sulfate, calcium sulfate with 20 wt% bioglass, and calcium sulfate with 40 wt% hydroxyapatite and found that CSH containing bioglass exhibited the highest compressive strength after setting for 7 days (18.1 ± 0.8 MPa), followed by pure CSH (16.5 ± 3.1 MPa) and CSH containing HA (7.3 ± 0.6 MPa). However, the increase in compressive strength occurred at the cost of prolonging the setting time and accelerating the degradation rate. After 28 days of soaking in SBF, 83.3% of the CSH/bioglass compound, 61.5% of the pure CSH, and 100% of the CSH/HA compound were degraded. In addition, the final setting time increased from 31 min for pure CSH to 57 min for CSH/bioglass and 51 min for CSH/HA. The mechanism of increasing compressive strength by adding calcium silicate into calcium sulfate was proposed as follows: (1) calcium silicate hydrate particles tend to fill the micropores between the calcium sulfate dihydrate crystals, in turn reducing the microporosity of the bone cement; and (2) the continuous hydration of tricalcium silicate results in the progressive polymerization of the calcium silicate hydrate gels and the development of a solid network, which could further fill the vacant regions within the composite cement, reduce the porosity of the material after prolonged setting time, and enhance the long-term compressive strength of the composite cement (46).

In addition, some additives can decrease the strength of calcium sulfate due to the reduction in the contact points between particles and weakening of adhesion forces (2). The impact of hydroxyapatite on the compressive strength of calcium sulfate is controversial. In vitro studies by Nilsson et al. (56) found that the strength decreased with increasing HA content because of the absence of reaction or chemical bonding between calcium sulfate and HA. SEM showed that the two phases are only held together by the CSD matrix. It can be inferred that HA is merely mechanically incorporated into the crystal structure and does not contribute to binding the material together. However, great achievements have been made in the clinical application of the mixture of calcium sulfate and HA, especially in the treatment of elderly patients with osteoporotic spinal fracture through percutaneous vertebroplasty (74).

## Wound drainage

In clinical applications, delayed wound healing or wound drainage is a recognized complication, especially in subcutaneous bones such as the tibia and ulna or when larger volumes of calcium sulfate are used (75). Friesenbichler et al. (76) originally attempted to evaluate the efficacy of calcium sulfate/TCP on the resorption profile and bone healing in 40 patients with bone defects in a prospective study, but the study was stopped as a result of serious complications after enrollment of the first 31 patients. In the report, 16% of patients had wound-related complications. In all, 2 patients experienced delayed wound healing due to sterile inflammation adjacent to the implants, 1 patient suffered from moderate-to-severe skin damage in the area of the scar and needed revision surgery, and 2 other patients developed inflammatory cystic formations in the soft tissues with sizes up to 15 cm. The authors did not support this type of bone substitute used in the treatment of bony defects. Saadoun et al. (77) reported that all 3 patients with spinal tumors had severe complications after cervical surgery. Of these patients, 2 patients suffered from wound infection and skin breakdown, and 1 patient experienced delayed pharyngeal perforation because of inflammatory destruction induced by adjacent calcium sulfate/TCP implants. In addition, the authors investigated the incidence of wound breakdown from 10 orthopedic surgeons in different units that used sulfate/TCP at least once as a bone filler during non-spinal surgery, finding that 40% of orthopedic surgeons experienced wound breakdown/purulent discharge.

With the increasing use of calcium sulfate in clinical application, wound problems have become a more concerning topic, compelling clinicians to identify the possible causes or mechanisms. Currently, the possible causes of this problem still include rapid resorption and subsequent low pH values. Previous studies have noted that the degradation products (such as sulfate radical ions) of calcium sulfate decrease the pH and produce an acidic microenvironment, which might lead to an inflammatory reaction with host tissues *in vivo* (67). The early use of pure calcium sulfate has revealed the disadvantage of delayed wound healing and osteolytic processes during resorption by pH lowering (23). An acidic microenvironment has been implicated in causing some local wound issues, with serous ooze in a proportion of cases (78–80), although this seems to be self-limiting (81). In addition, we hypothesized that the placement of calcium sulfate alters the osmolality of the operating field, leading to the movement of water out of cells with the accumulation of fluid and wound drainage (80–82).

However, numerous of studies have revealed that changes in the extracellular fluid pH of the local biological microenvironment can profoundly affect cell metabolism and function (83, 84). Walsh et al. (85) reported that the potential mechanism for inducing osteogenesis at low pH is that local acidity leads to the demineralization of adjacent bone, releasing matrix-bound BMPs and resulting in a stimulatory effect. We can infer that a local acidic environment caused by the dissolution of calcium sulfate has two functions: positive effects on new bone formation and negative effects on incision healing. How to reduce implant-related complications without jeopardizing osteogenic properties is still controversial. Considering the causes of complications, a possible method to inhibit wound-related complications should be to incorporate alkaline additives or materials with low degradation performance. The former can buffer the low pH caused by calcium sulfate absorption, and the latter can avoid a great release of sulfate ions to surrounding soft tissues in a short time after implantation.

Previous studies have shown that the degradation products of calcium carbonate and silicate are alkaline substances, which can neutralize the acidic environment produced by the dissolution of calcium sulfate. D. Pförringer et al. (43) demonstrated that the rationale for the use of calcium carbonate as an additive is its ability to delay degradation of CS and buffer the pH value in the implant region, thus counteracting healing problems of the tissue in a rabbit model. Feng et al. (86) pointed out that silicates neutralize the acidic degradation byproducts and stabilize the pH of the surrounding environment. Lin et al. (30) investigated the effect of calcium silicate on the pH of CSH composites and found that the pH values of the composites containing calcium silicate rapidly increased with an increase in setting time, achieving a maximum (pH 8.8–9.3) at 24 h, and thereafter remained stable. In contrast, the pH value of pure CSH was reduced to 7.0 within 8 h and then maintained a small change. The results of an *in vitro* study by Wang et al. (84) suggested that the addition of calcium silicate with or without Sr into calcium sulfate cement may have caused a pH fluctuation toward high pH values within the initial 7 days, and it became relatively stable for the remaining soaking procedure. Perhaps this is of great benefit for osteogenic cell activity and bone regeneration. Ding et al. (87) investigated the effect of magnesium–calcium silicate on the pH value of the solution and found that the pH of pure CSH decreased sharply from 7.4 to 6.96 within 14 days and further decreased to 6.89 after soaking for 84 days. However, the pH for those containing 20 wt% and 40 wt% magnesium–calcium silicate showed mild changes (from 7.4 to 7.22 and from 7.4 to 7.26, respectively) within 14 days and gradually increased to 7.28 and 7.34, respectively, after soaking for 84 days. The author concluded that magnesium–calcium silicate could neutralize the acidic degradation products of calcium sulfate and prevent the pH from dropping. In addition, magnesium–calcium silicate markedly improved the attachment, proliferation, and differentiation of cells on the surface of the bone graft. However, the authors also found that the degradation ratio increased, and the compressive strength decreased with increasing m-MCS content, suggesting that calcium magnesium silicate is not an ideal additive for calcium sulfate.

In addition, the effect of calcium phosphate on pH has also been reported. Chen et al. (67) studied the physical and chemical properties of a mixed power containing tetracalcium phosphate (TTCP)/dicalcium phosphate anhydrous DCPA/calcium sulfate hemihydrate with a weight of 2.69:1:4.51 and found that the pH value of the immersion solution decreased from 6.5 for 1 day to 6.2 for 3 days due to the dissolution of CSH and formation of CSD. Within 7 days, the pH value of the solution started to increase, probably due to the continual dissolution of TTCP and DCPA. After 7 days, the average pH value became 6.6 and remained stable until 28 days. After 42 days, the average pH value decreased to 6.3, probably as a result of the formation of HA and the dissolution of CSD. All pH values were easily found to be in the weakly acidic range from 6.2 to 6.6 during the entire immersion process when calcium phosphate was incorporated into the CSH powder.

At present, it is generally believed that the wound problem could be related to the acidic environment caused by the degradation products of calcium sulfate. From the aspect of production technology, additives that can increase the pH value or slow the release of acid, such as sulfate ions, should be added.

## Hypercalcemia

Hypercalcemia resulting from calcium sulfate is a rare but severe side effect, and alterations in mental status are the most common clinical symptoms. In addition, a few patients may have renal failure or acute encephalopathy secondary to hypercalcemia or antibiotic toxicity (88, 89). Hypercalcemia was first described in a canine model, but elevated serum calcium levels were not sustained, and no symptoms were noted in the 1950s (41). In 2005, the FDA warned that calcium sulfate may cause hypercalcemia based on an adverse reaction report about transient hypercalcemia following the placement of vancomycin-infused CSBs in a patient undergoing hip arthroplasty (90). However, it was not until 2015 that the first clinical case of hypercalcemia following the use of absorbable calcium sulfate in hip arthroplasty was reported and published by Carlson et al. (88). Another study in the same year by Kallala et al. (91) demonstrated that 3 of 15 patients with a periprosthetic joint infection developed hypercalcemia, with 1 patient developing symptoms of altered mental status, who received CS-containing antibiotics in revision arthroplasty.

The reasons for hypercalcemia resulting from the use of calcium sulfate are unclear (82). One hypothesis is a dose-dependent relationship between the volume of calcium sulfate used and hypercalcemia (91). Kallala et al. (93) evaluated complications of CS in a cohort of 755 patients with revision arthroplasty: 41 patients developed hypercalcemia, of whom 2 exhibited symptoms and received treatment with intravenous fluids and a single dose of bisphosphonate. The authors found that the incidence of hypercalcemia increased with the dosage of calcium sulfate. Although the conclusion was drawn from the largest number of cases (93), no direct correlation between hypercalcemia and calcium sulfate concentration was found in subsequent studies. A recent study by Jiang et al. (94) investigated the relationship between the incidence of hypercalcemia and the dosage of calcium sulfate in patients who received local CS implantation for the management of posttraumatic osteomyelitis and found that none of the patients had hypercalcemia, and no significant links were identified between CS volume and postoperative calcium levels. In another recent case report, Vora et al. (88) demonstrated that the volume of CS placed was not related to hypercalcemia. To date, there is no evidence that a large amount of calcium sulfate can increase the risk of transient hypercalcemia.

Another possibility is that the sudden release of Ca^2+^ into body fluid due to rapid degradation of CS leads to locally increased concentrations of calcium, which then enters the blood vessels in a short period (92). Carlson et al. (89) reported that the complete absorption time of CS is only 5 days after surgery in patients who underwent surgical placement of calcium-based beads into a hip arthroplasty, which could be the cause of hypercalcemia. Challener et al. (95) found accelerated degradation of calcium sulfate beads through a CT scan of the lower extremity, which made the authors highly suspicious that hypercalcemia might be related to the degradation rate. Interestingly, patients with hypercalcemia are those with periprosthetic joint infections. In addition, Table 1 shows that all calcium sulfate materials used in these studies were mixed with antibiotics in advance. We can infer from the above that antibiotics coated with calcium sulfate damage the interlocking structure and reduce the adhesion forces between calcium sulfate particles, which may greatly increase the degradation rate and lead to the release of large amounts of calcium ions and antibiotics into body fluids (89). At present, there is no report about this complication in patients with spinal fusion, bone defect reconstruction, and bone non-union.

**Table 1:** Clinical application of calcium sulfate and antibiotic mixed bone material.

Author	State (time)	Gender	Age	Disease	Site	Number	Product	Antibiotic
Vora and Ali (89)	USA (2019)	Female	58	Infection after arthroplasty	left hip	1	–	–
Kallala and Haddad (92)	UK (2015)	–	64.8 (41–83)	Infection after arthroplasty	Revision arthroplasty	3/15	Stimulan	1 g vancomycin 240 mg Gentamicin per 20 g CS
Magdaleno and McCauley (93)	USA (2019)	Female	61	Infection after arthroplasty	Right knee	1		tobramycin and vancomycin
Kallala et al. (94)	UK (2018)	–	–	after arthroplasty	456 knee and 299 hip	41/755	Stimulan	–
Challener and Abu Saleh (96)	USA (2019)	Female	90	Infection after internal fixation	Left trochanteric fracture	1	–	7.2 g gentamicin 6 g of vancomycin

While few studies have reported methods to overcome this defect, it can be observed that controlling the degradation rate can prevent hypercalcemia. The method for controlling the degradation rate has been introduced above and will not be repeated here. Except for the defects of calcium sulfate itself, clinicians should be reminded to pay attention to the mental state of patients and monitor serum calcium and creatinine levels within 72 h after calcium sulfate implantation (95). Patients at risk of developing hypercalcemia, including those with pre-existing hypercalcemia, renal impairment, critical illness, prolonged immobilization, and parathyroid disorders, should be approached with additional caution, and alternative strategies should be considered.

Another way to prevent hypercalcemia is through the addition of hydrophobic material, which renders the originally hydrophilic calcium sulfate more hydrophobic, and ensuring that no relevant quantities of calcium ions are dissolved in the blood (11). Hydrophobic materials generally refer to organic materials, which are not addressed in this study. Further studies concerning the effect of organic materials on the properties of CS are needed.

## Osteogenic activities

Calcium sulfate pellets or bone cement have been clinically used for more than a century because of their biocompatibility and biodegradability. At present, there are two possible mechanisms of osteogenesis (96). The first is that the release of calcium and sulfur ions in the biological environment results in apatite formation, and elevated calcium ion concentrations may increase osteoblastic activity, including osteoblastic genesis and differentiation (96–98). It can promote bone regeneration, that is, improve the mechanical stability of bone. The second is that a local drop in pH during calcium sulfate dissolution causes surface demineralization of the surrounding bone and subsequent exposure of growth factors such as transforming growth factors and bone morphogenetic proteins, thus stimulating new bone formation (88, 99). In addition, mechanical stability is also necessary in the process of bone healing (24). In 1892, Dreesmann et al. (100) first reported the use of calcium sulfate to treat large bone defects. Subsequent reports showed good results with complete bone regeneration (101, 102). Peltier (41) reported on 20 patients and concluded that calcium sulfate showed good biocompatibility and did not cause any complications even in infected cavities. In addition, Siemund et al. (103) first reported the use of calcium sulfate-based bone cement to replace PMMA in the treatment of osteoporotic vertebral fracture. Then, Rauschmann et al. (104) used calcium sulfate/hydroxyapatite as a substitute for PMMA to treat 15 patients with osteoporosis vertebral fractures involving 16 vertebrae and found that pain relief was maintained over 18 months and that no adjacent fractures were observed. Other studies have also proven the efficacy of calcium sulfate cement in the treatment of osteoporotic or traumatic vertebral fractures by percutaneous vertebroplasty (74, 105).

However, the osteogenic activity or intrinsic osteoinduction of calcium sulfate is still controversial (106). A prospective randomized controlled trial by D'Agostino et al. (107) did not demonstrate a beneficial osteogenic effect of the calcium sulfate/DBM complex in the treatment of this category of distal radial fractures. Yu et al. (108) reported the poor results and early failure of using CaSO_4_/CaPO_4_ composite grafts for the treatment of osteonecrosis of the femoral head. The author did not recommend continuing the application of such materials. The results were completely opposite to those of Civinini et al. (109), who found that the use of CaSO4/CaPO4 composite grafts could relieve hip pain and prevent the progression of osteonecrosis of the femoral head. Additionally, Turner et al. (33) identified that calcium sulfate merely provided an osteoconductive substrate. Furthermore, Beuerlein et al. (110) clearly elucidated that the shortage of calcium sulfate was neither osteoinductive nor osteogenic.

Although the osteogenesis mechanism has been reported by researchers, several poor properties of calcium sulfate for new bone growth were not ignored. First, the voids between implants and surrounding bone by rapid absorption made calcium sulfate lose its osteogenesis ability, which is an indisputable fact. In addition, the implant cannot provide a long enough mechanical environment for osteogenesis, which has proven to be possible by Yu et al. (97), who pointed out that although the graft may have enough strength initially, its structural integrity would be lost in the very early postoperative period, which damages the mechanical environment of osteogenesis during new bone formation. Third, the implant cannot form chemical bonds between the calcium sulfate grafts and bone tissue, which may influence the differentiation, metabolism, and growth of osteoblasts (111, 112). Fourth, the lack of porosity has been shown to decrease the osteoconductivity of calcium sulfate, having an adverse impact on creeping substitution (113, 114). Therefore, calcium sulfate is rarely used alone in clinical applications (115).

To promote osteogenesis, additives should be selected carefully. Any additives that can deteriorate the degradation rate or decrease the compressive strength can affect new bone growth (36), which also explains the poor effect of the CS/DBM complex (116, 117). Another study by Kelly (68), in comparing the effect of pure calcium sulfate and the calcium sulfate complex on osteogenesis, also proved that DBM or autogenous bone may decrease new bone growth, finding that patients treated with calcium sulfate alone had 99% pellet resorption and 98% bone growth at 6 months and 100% pellet resorption and 99% bone growth into the defect at 1 year. Patients who were treated with the calcium sulfate complex had 98% pellet resorption and 83% bone ingrowth at 6 months and 100% pellet resorption and 92% bone ingrowth at 1 year.

Additives that can improve the properties of calcium sulfate can promote osteogenesis. Previous studies showed that the role of silicate may extend beyond the ability to form an HAP surface layer that bonds to bone, and silicon dissolved into the body fluid could upregulate osteoblast proliferation, differentiation, and bone-related gene expression (118). In addition, an appropriate Si concentration is beneficial to collagen type I formation (119). Hao et al. (49) found that soluble silicate contributed to the formation of an amorphous SiO2-rich surface layer, which attracted the migration of Ca^2+^ and PO4^3−^, forming a calcium phosphate (CaeP)-rich layer on silicate-based materials that ultimately formed a hydroxyapatite (HAP) layer after crystallization of the CaeP layer; HAP could bond to living bone. Urban et al. (57) investigated the effect of calcium phosphate on the osteogenesis of calcium sulfate in a critical-sized canine bone defect model. They found that the area fraction of new mineralized bone was twofold greater in defects treated with the composite graft material than in defects treated with conventional CaSO4 pellets and in normal bone at 13 weeks after implantation. The authors observed that the new bone layered on the surfaces of the residual DCPD matrix and TCP material, forming a concentric ring-like pattern. The addition of calcium carbonate can promote the formation of carbonated apatite and induce blood vessel growth or new bone formation (120). D. Pförringer (43) reported that the addition of calcium carbonate can delay the degradation of the implants and result in synchronization of the ingrowing bone with biomaterial resorption but does not have negative effects on biocompatibility.

In terms of improving osteogenesis, the purpose of adding inorganic salt is mainly to decrease the resorption rate and provide a stable microenvironmental pH, which promotes the deposition of calcium salt and metabolic activity and contributes to cell adhesion, proliferation, and differentiation of osteoblasts (118, 119). In fact, the osteogenesis of calcium sulfate is not as bad as the values in the literature, so it is very important to choose the right indication. With the development of preparation technology for the calcium sulfate complex, its osteogenesis will improve.

## Conclusions

Calcium sulfate is a relatively inexpensive, widely available synthetic bone substitute. However, pure calcium sulfate is rarely used in the clinic because of its poor compressive strength and rapid degradation rate and subsequent complications, such as wound drainage, hypercalcemia, and osteogenic activities. Calcium sulfate-based composites are the main clinical application form, in which inorganic salt is the main additive to improve the physical and chemical properties of calcium sulfate. At present, common inorganic composite materials include calcium phosphate, calcium carbonate, and calcium silicate.

(1) Calcium carbonate has a positive effect on reducing the degradation rate, improving the osteogenic ability, and buffering the acidic environment, but this is at the expense of compressive strength (119). Calcium carbonate can only be used to fill small bone defects or non-weight-bearing parts and treat bone defects as a drug release carrier (121).
(2) Calcium silicate, including tricalcium silicate and dicalcium silicate, has the ability to stimulate new bone formation, decrease the degradation rate, and increase the compressive strength of calcium sulfate (118). Bioglass containing silicon dioxide can increase compressive strength at the cost of prolonging setting time and accelerating the degradation rate (73).
(3) Calcium phosphate, including hydrogen orthophosphate and tricalcium phosphate, has been shown to significantly accelerate calcium phosphorus deposition, decrease the degradation rate, and reinforce the CSH composite at different time stages *in vivo* and *in vitro* (122). However, the effect of hydroxyapatite, as a form of calcium phosphate, on compressive strength is still controversial (56).
